# Editorial: From cobots to human-robot synergy–overview and future trends

**DOI:** 10.3389/frobt.2023.1270373

**Published:** 2023-08-18

**Authors:** Anna Valente, Oliver Avram

**Affiliations:** Automation, Robotics and Machines Laboratory, Department of Innovative Technologies, Institute of Systems and Technologies for Sustainable Production, University of Applied Sciences and Arts of Southern Switzerland (SUPSI), Viganello, Switzerland

**Keywords:** human-swarm interaction, human-robot interaction, human cognitive state assessment, harsh environments, exoskeleton, system behavior

In recent years, technological advancements have paved the way for innovative solutions that integrate human operators with autonomous systems. The Research Topic “*From Cobots to Human-Robot Synergy*” delves into this fascinating dimension of human-machine collaboration, where robots and humans work together as a team to achieve common goals. Three distinct works shed light on various facets of this rapidly evolving topic, emphasizing the critical importance of adaptive autonomy, deliberative capabilities, and future-oriented support technologies in enhancing human-machine collaboration. Each paper contributes unique insights into the development of teaming frameworks promoting the role of robots from mere tools to efficient teammates able to learn from interactions, experiences and specific contexts and to adapt their behavior to suit the preferences and needs of their human partners.

The first article of this Research Topic (Hussein et al.) introduces the captivating world of swarms for urban search, rescue and cyber defense, with a keen focus on the critical coupling between humans and the swarm. The authors delve into five key aspects necessary for an adaptive agent to operate effectively, including mission objectives, interaction, mission complexity, automation levels, and human states. To ensure seamless interaction between humans and the swarm during missions, coordination is vital. Their approach employs an adaptive agent at the interface, skilfully monitoring and managing the system’s entities. This agent dynamically adjusts the level of autonomy based on mission states, using adaptive autonomy to strike the perfect balance between human workload and autonomous system performance. Furthermore, the research addresses the cognitive state of users in the human-swarm interaction. By assessing real-time human cognitive states like workload, fatigue, and attention, the system is enabled to make timely adjustments, ensuring safety and efficiency. Translating these cognitive states into meaningful guidance empowers the system to adapt effectively.

With an alternative approach accounting for the cognitive load and emotional states, Avram et al. capitalizes on the teaming of humans and mobile robots by cultivating intelligent robot behaviors to alleviate stress factors and mitigate human exposure to hazardous tasks. The authors propose a deliberative framework for harsh working environments, aiming to proactively identify possible human errors and at-risk behaviors that could lead to accidents or compromise operational efficiency and reliability. The framework interprets these factors as primary behavioral cues affecting the robot’s decisional process and provides a well-organized integration of an extended set of robotic capabilities, including navigation, human activity perception, HRI, emotion recognition, deliberation, and planning and execution of tasks within MRO settings. The central goal is to trigger adapted behavioral responses that not only improve operational efficiency but also significantly enhance the occupational health and welfare of humans working in challenging environments. Exploring further the deliberative aspects, the framework relies upon three behavioral dimensions. First of all, it identifies potential hazards and safety issues in the current working situation involving the human. Next, it assesses the emotional state of the human, considering the impact of excessive psychological burden on task performance. Lastly, it proposes alternative routes to ease the difficulty of tasks, fostering a shared human-robot plan for optimal task execution.

Unlike the first two frameworks which emphasize the potential of separate robotic teammates to complement and enhance the abilities of human teammates, Ott et al. discusses human-machine teaming dynamics from the perspective of direct-contact support technologies such as exoskeletons. The main motivation stems from the lack of scientific investigation and standardized tools to qualify exoskeletons as adaptable support technologies. Their framework aims at laying groundwork for the future-oriented qualification of exoskeletons and represents a novel and holistic approach to describing the support characteristics of these physical support technologies. Furthermore, it emphasizes the importance of user-individual and context-dependent adaptivity in exoskeletons’ interaction with operators. The need for adaptive systems arises from the ever-changing conditions and complexity of human beings and activities and the work carried out bridges this gap by enabling the description of existing support characteristics and defining future system behaviour. Exoskeletons, with their user-specific and context-dependent adaptivity, hold significant potential for providing targeted support in manual work processes.

All papers collectively highlight the vital aspects of adaptive autonomy, deliberative frameworks, and future-oriented support technologies in achieving effective and harmonious human-robot collaboration. From swarm systems to mobile robots and exoskeletons, the presented frameworks paint a compelling picture of how human-robot synergy can be harnessed to unleash the full potential of collaborative robotics. As researchers and practitioners delve deeper into these areas, the journey towards a future where humans and robots work hand in hand in perfect harmony continues to accelerate, promising a world of enhanced productivity, safety, and wellbeing.

